# Recombinant Trimeric HA Protein Immunogenicity of H5N1 Avian Influenza Viruses and Their Combined Use with Inactivated or Adenovirus Vaccines

**DOI:** 10.1371/journal.pone.0020052

**Published:** 2011-05-31

**Authors:** Shih-Chang Lin, Ming-Hsi Huang, Pei-Chun Tsou, Li-Min Huang, Pele Chong, Suh-Chin Wu

**Affiliations:** 1 Institute of Biotechnology and Department of Life Science, National Tsing Hua University, Hsinchu, Taiwan; 2 Vaccine Research and Development Center, National Health Research Institutes, Zhunan, Miaoli County, Taiwan; 3 Department of Pediatrics, National Taiwan University Hospital, National Taiwan University, Taipei, Taiwan; Johns Hopkins University - Bloomberg School of Public Health, United States of America

## Abstract

**Background:**

The highly pathogenic avian influenza (HPAI) H5N1 virus continues to cause disease in poultry and humans. The hemagglutinin (HA) envelope protein is the primary target for subunit vaccine development.

**Methodology/Principal Findings:**

We used baculovirus-insect cell expression to obtain trimeric recombinant HA (rHA) proteins from two HPAI H5N1 viruses. We investigated trimeric rHA protein immunogenicity in mice via immunizations, and found that the highest levels of neutralizing antibodies resulted from coupling with a PELC/CpG adjuvant. We also found that the combined use of trimeric rHA proteins with (a) an inactivated H5N1 vaccine virus, or (b) a recombinant adenovirus encoding full-length HA sequences for prime-boost immunization, further improved antibody responses against homologous and heterologous H5N1 virus strains. Data from cross-clade prime-boost immunization regimens indicate that sequential immunization with different clade HA antigens increased antibody responses in terms of total IgG level and neutralizing antibody titers.

**Conclusion/Significance:**

Our findings suggest that the use of trimeric rHA in prime-boost vaccine regimens represents an alternative strategy for recombinant H5N1 vaccine development.

## Introduction

Influenza viruses trigger seasonal disease epidemics and potential pandemics, both with mild-to-severe consequences for human and poultry populations [1]. Influenza type A virus, a member of the *Orthomyxoviridae* family, consists of single-stranded eight-segment negative-sense genomic RNAs, helical viral ribonucleoprotein (RNP) complexes (RNA segments NP, PB2, PB1 and PA), three viral envelope proteins (hemagglutinin [HA], neuraminidase [NA], and M2 ion channel), and a maxtir (M1) protein. Influenza A viruses are further classified into 16 HA (H1–H16) and 9 NA (N1–N9) serotypes based on the antigenic characteristics of HA and NA envelope glycoproteins [2].

In aquatic birds, the 16 HA and 9 NA influenza A virus subtypes are not disease triggers [2]. In contrast, highly pathogenic avian influenza (HPAI) viruses such as H5N1, H7N3, H7N7 and H9N2 can result in severe diseases with mortality in poultry, and occasionally in human populations [3]. H5N1 was the main virus in the first human outbreak in 1997; it emerged again in 2003, and has continued to cause disease in poultry and humans. Between 1997 and 2010, human HPAI H5N1 resulted in rare and sporadic, but often severe and fatal human infections in Asia, the Middle East, Eastern Europe, and Africa. The mortality rate for the 520 cases reported during that period was 59% [4].

HA, a major envelope glycoprotein, is a major target for the development of influenza vaccines. Recombinant HA (rHA) proteins have been developed as a subunit vaccine against H5N1 infection. The rHA vaccine approach is an attractive alternative for vaccine manufacturing because it removes the need for egg-based or cell-based H5N1 influenza virus vaccine production, thus eliminating the associated requirement for 2+ or 3 biosafety levels for facilities and equipment. Several research teams have reported that neutralizing antibody titers against the H5N1 virus can be induced in mice, chickens, and ferrets via rHA proteins produced from insect cells [5], [6], [7], mammalian cells [7], [8], [9], plant cells [10], [11] and *E. coli* [6], [12], [13], [14], [15]. For this study we used baculovirus-insect cell expression to obtain rHA proteins from two HPAI H5N1 strains: KAN-1 and Anhui. The rHA proteins were engineered to form trimers using additional sequences from the leucine zipper GCN-pII [16] fused at the C-terminal end. Mice immunized with trimeric rHA proteins coupled with Alum, CpG, Alum/CpG, PELC [17], or PELC/CpG are capable of eliciting HA-specific IgG responses and neutralizing antibodies. In addition, we combined trimeric rHA proteins with an inactivated H5N1 vaccine virus, or recombinant adenovirus (rAd-HA) encoding full-length HA sequences of HPAI H5N1 viruses as part of a prime-boost immunization strategy aimed at enhancing elicited antibody responses against homologous and heterologous H5N1 virus clades.

## Results

### Trimeric rHA protein construction, purification, and characterization

**Figure 1:**
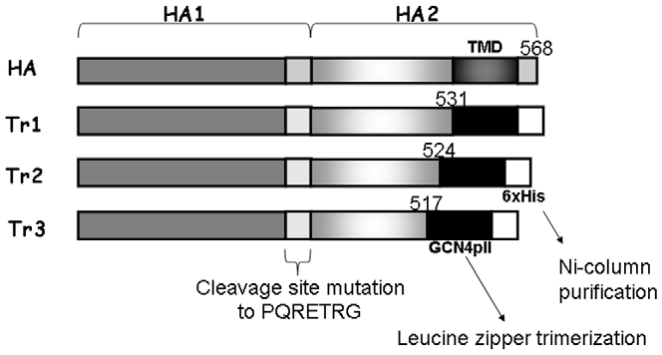
HA protein constructs. Vectors encoding three truncated forms of soluble trimeric HA (Tr1, Tr2, Tr3) were constructed using the baculovirus expression vector pFastBac 1. In these constructs, the polybasic cleavage site between HA1 and HA2 was changed from PQRRRKKRG to PQTRG to prevent cleavages. Transmembrane and cytoplasmic domains at the C terminus of full-length HA were deleted and replaced with a leucine zipper GCN4-pII sequence (MKQIEDKIEEILSKIYHIENEIARIKKLIGEV) for trimerization. An His-tag was added for purification.

**Figure 2:**
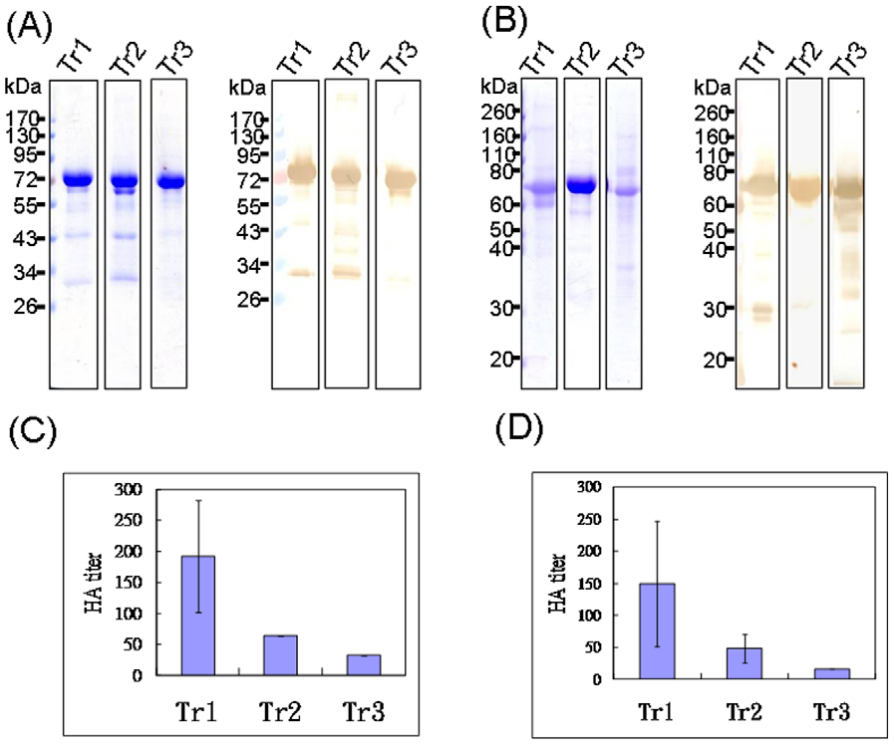
HA protein purification. Trimeric HA proteins were obtained via recombinant baculoviruses encoding H5N1 HA sequences from culture supernatant. Three truncated forms each of HA proteins from KAN-1 (A) and Anhui (B) strains were purified using metal affinity chromatography with Coomassie blue staining (left). Purified proteins were confirmed by western blotting (right) using anti-6xHis antibody. Purified HA proteins from KAN-1 (C) and Anhui (D) strains were digested with trypsin to confirm that the HA with a cleavage site mutation could be cleaved into HA1 (detected by polyclonal anti-H5HA antibodies, left) and HA2 (detected by polyclonal anti-6xHis antibodies, right).

**Figure 3:**
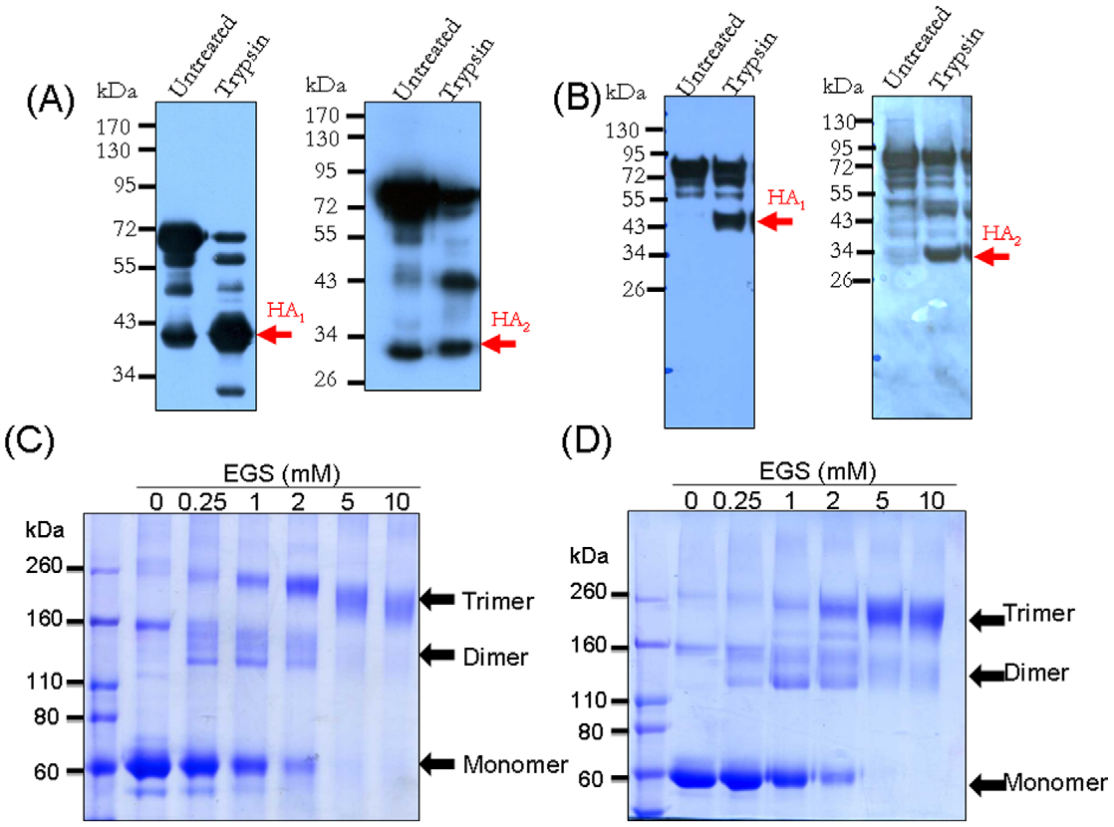
HA protein characterization. Trimers of the Tr1 forms of HA proteins from KAN-1 (A) and Anhui (B) strains were examined using the homobifunctional and cleavable cross-linking reagent ethylene glycol-bis (EGS). Tr1 monomers were cross-linked into a dimer and then into a trimer as EGS concentration increased. Turkey red blood cells were used to test the hemagglutination activity of purified KAN-1 (C) and Anhui (D) HA. Of the three truncated forms, Tr1 displayed the highest HA titer level.

We used a baculovirus-insect cell expression system to generate three truncated forms of rHA (Tr1, Tr2, Tr3), with the transmembrane and cytoplasmic domains at the C terminus of full-length HA sequences replaced with the GCN4pII sequence KQIEDKIEEILSKIYHIENEIARIKKLIGEV and a His tag (Fig. 1). The polybasic cleavage site between HA1 and HA2 was changed from PQRRRKKRG to PQTRG to prevent unwanted cleavages in baculovirus-infected insect cells. We obtained rHA proteins from the culture supernatants of Sf9 cells infected with the recombinant baculoviruses. The three truncated rHA forms of the KAN-1 and Anhui strains were purified using Ni-NTA agarose chromatography (Fig. 2A–B). Hemagglutination of the three forms was tested for using turkey red blood cells; results indicate that the Tr1 proteins retained the highest HA titers compared to the Tr2 and Tr3 forms (Fig. 2C–D). We also treated the Tr1 rHA proteins of KAN-1 and Anhui strains with trypsin, revealing HA protein cleavage into HA1 and HA2 subunits (Fig. 3A–B). KAN-1 and Anhui rHA trimeric structures were evaluated by treatment with ethylene glycol-bis (EGS), a homobifunctional and cleavable cross-linking reagent previously used to analyze the trimeric form of HIV-1 gp120 [18]. According to our results, Tr1 monomers were cross-linked with a dimer, and then with a trimer, indicating that most of the rHA proteins of the KAN-1 and Anhui strains are trimers (Fig. 3C–D).

### Trimeric rHA protein immunogenicity

**Figure 4:**
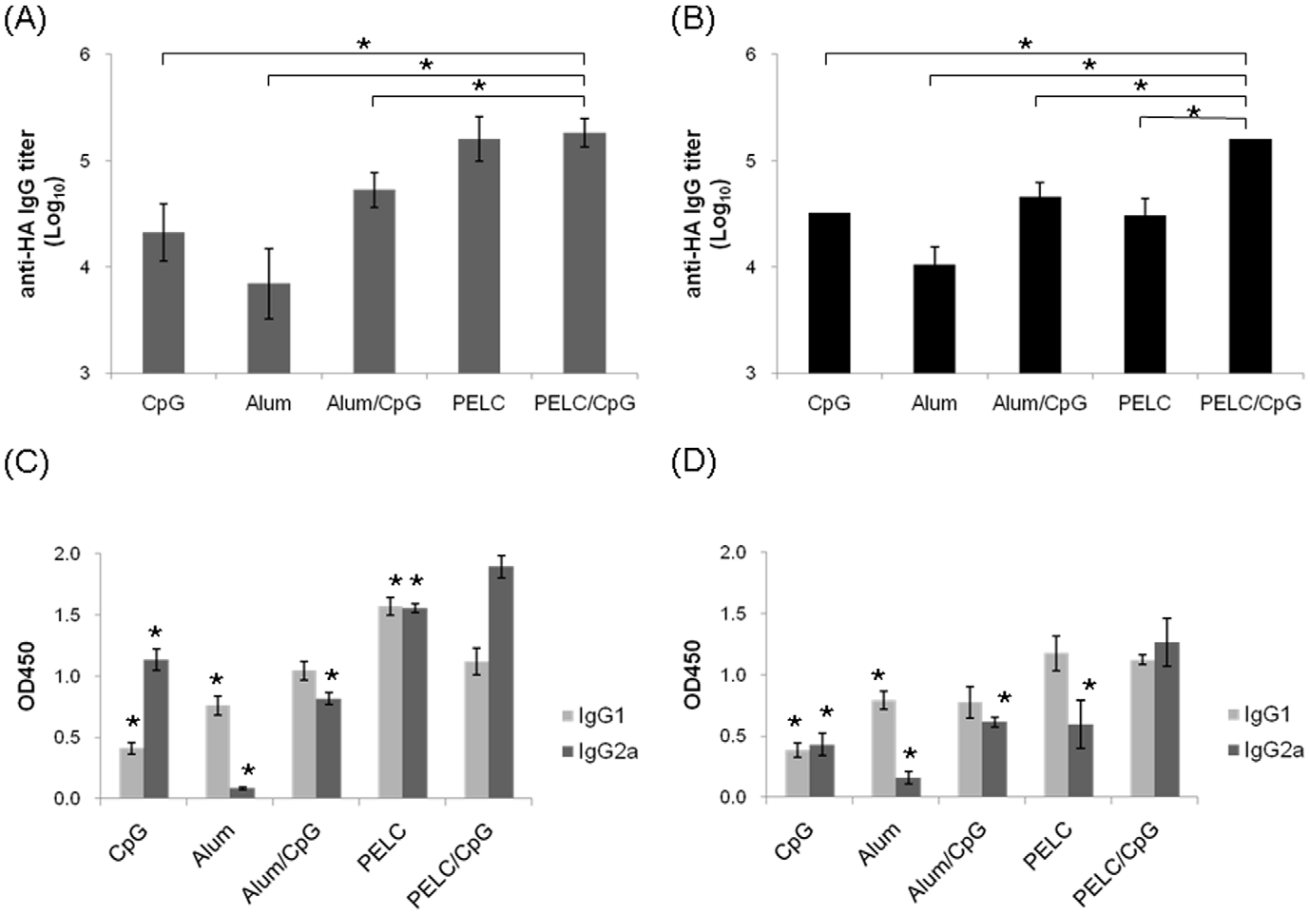
Antibody responses elicited by HA proteins in immunized mice. BALB/c mice were intramuscularly inoculated with two doses of HA protein (15 µg) coupled with one of five adjuvant formulations. Total IgG antibody responses in mice immunized with KAN-1 (A) or Anhui (B) HA were evaluated 2 weeks following the second immunization. HA protein immunization coupled with PELC/CpG adjuvant induced the largest amount of IgG titers. IgG subclasses were detected using ELISA. PELC and PELC/CpG adjuvants coupled with KAN-1 (C) or Anhui (D) HA induced higher levels of IgG1 and IgG2a subtypes compared to the Alum or CpG adjuvants. All values are expressed as geometric mean with a standard error of the mean of five mice per group. Asterisk (*) indicates a statistically significant difference compared to the PELC/CpG group (p<0.05, Student *t* test).

**Figure 5:**
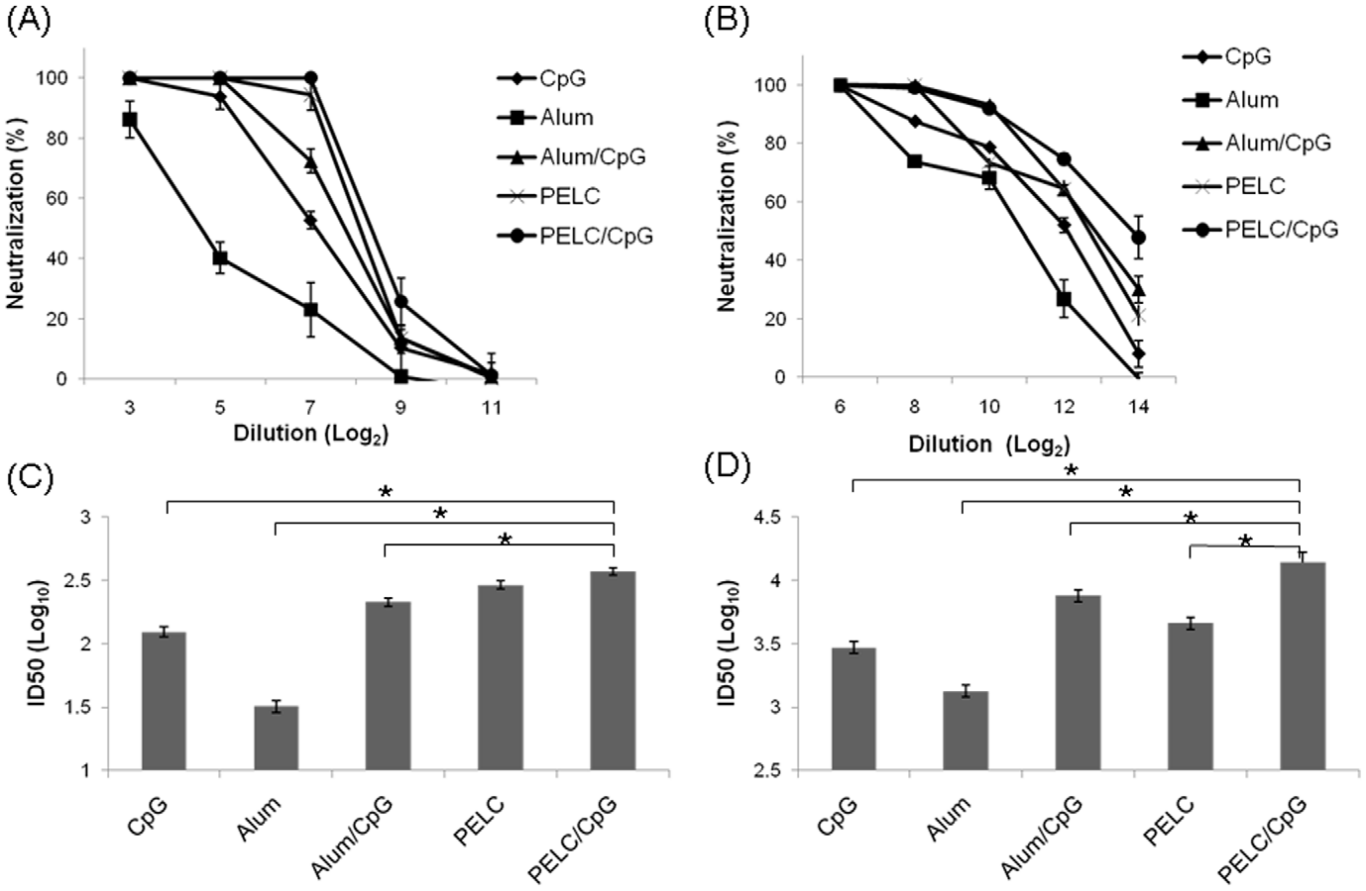
Neutralization against H5 pseudotyped particles in HA-immunized mice. Neutralization antibody titers were measured as reduction in luciferase activity of the H5HA-pseudotyped particle (H5pp) following the incubation of sera with H5 pseudotyped particles. p24 of H5pp (10 ng) was incubated with four-fold serial dilutions of serum for 1 h at 37°C and then transferred to MDCK cells. Luciferase assays were performed 48 h later. Dose-dependent neutralization curves were plotted against homologous KAN-1 (A) and Anhui (B) strains. Neutralization titers against homologous KAN-1 (C) and Anhui (D) strains and standard deviations were calculated using the ID50 program developed by John Spouge of the National Center for Biotechnology Information, National Library of Medicine, US National Institutes of Health. PELC/CpG elicited the highest level of neutralization titers, and Alum the lowest. Asterisk (*) indicates a statistically significant difference compared to the PELC/CpG group (p<0.05, Student *t* test).

To determine the immunogenicity of KAN-1 and Anhui trimeric rHA proteins, we intramuscularly immunized a group of BALB/c mice with 15 µg rHA per mouse, coupled with an adjuvant of Alum (300 µg/dose), CpG oligodeoxynucleotides (10 µg/dose), Alum/CpG, PELC (10%), or PELC/CpG. Two immunizations were given over a 3-week period. Anti-sera were collected 2 weeks after the second immunization, and used to evaluate elicited antibody responses. According to results from an ELISA coated with the trimeric rHA proteins of KAN-1 (Fig. 4A) or Anhui (Fig. 4B), mice immunized with either rHA protein plus the PELC/CpG adjuvant produced the highest levels of HA-specific total IgG titers. Immunization with trimeric rHA proteins plus PELC or PELC/CpG adjuvants also induced higher IgG1 and IgG2a subtype titers compared to trimeric rHA proteins plus Alum, CpG, or Alum/CpG (Fig. 4C–D). Neutralization curves against KAN-1 or Anhui H5pp indicate that immunization with the trimeric rHA proteins plus any of the adjuvant formulations that were investigated in this study induced neutralizing antibody responses in a dose-dependent manner (Fig. 5A–B). Corresponding log (ID-50) values against KAN-1 H5pp and Anhui H5pp are shown in Figures 5C–D. According to these results, trimeric Anhui rHA proteins were more immunogenic than those of the KAN-1 strain for all of the investigated adjuvant formulations. The PELC/CpG adjuvant was the most effective of the four adjuvant formulations in terms of enhancing antibody responses in mice immunized with trimeric rHA proteins.

### Combined use of trimeric rHA proteins with an inactivated or adenovirus vaccine for prime-boost immunization

**Figure 6:**
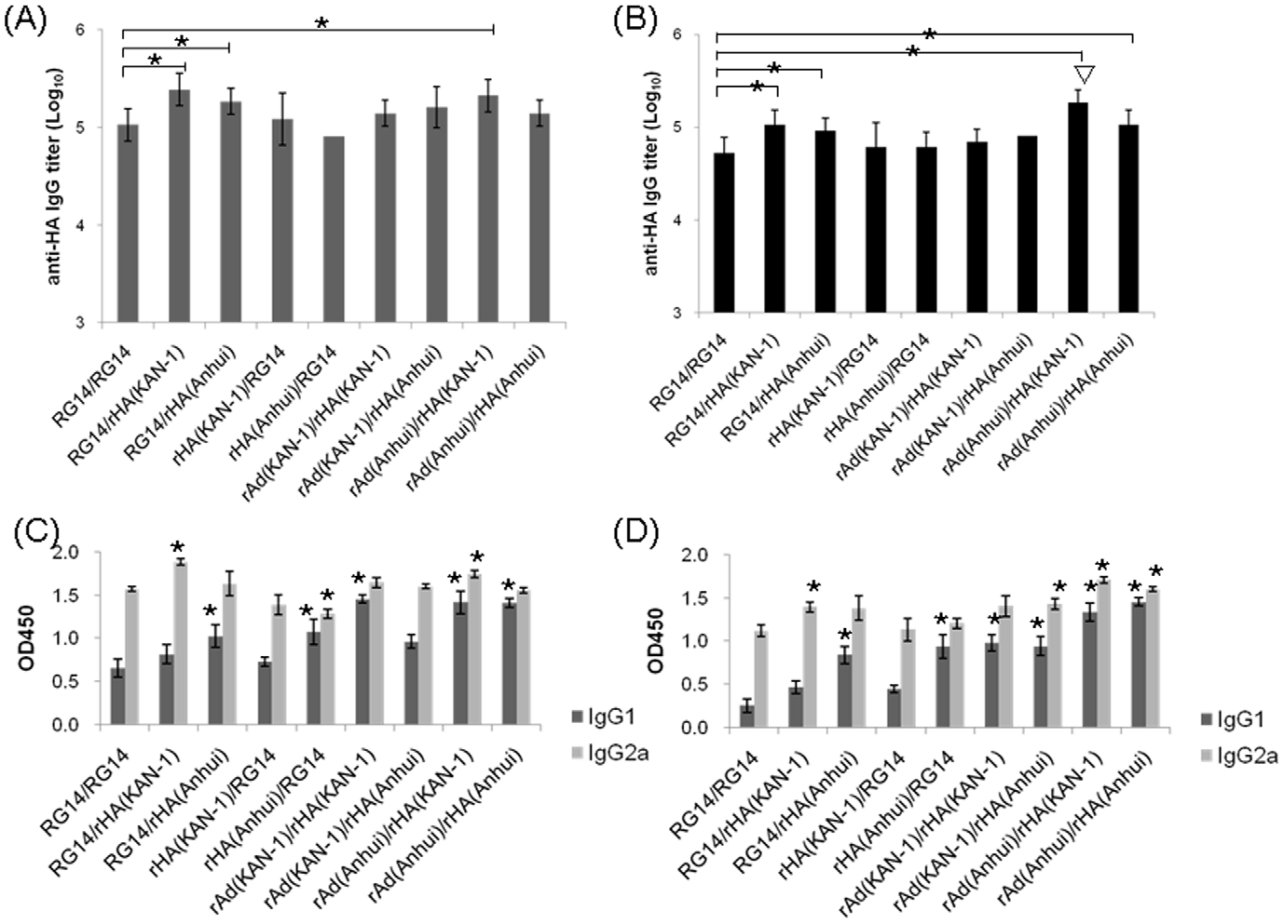
Antibody responses in mice with combined immunizations. Mice were immunized with trimeric KAN-1 or Anhui HA proteins in combination with inactivated H5N1 vaccine virus NIBRG-14 (clade 1), or a recombinant adenovirus encoding the full-length HA of KAN-1 [rAd(KAN-1)] or Anhui [rAd(Anhui)]. Total IgG titers from antisera against KAN-1 (A) and Anhui (B) were measured using ELISA; IgG1 and IgG2a against KAN-1 (C) and Anhui (D) were also determined. Values are expressed as geometric mean with a standard error of the mean of five mice per group. Asterisk (*) indicates a statistically significant difference compared to the double-dose of inactivated NIBRG-14 group (p<0.05, Student *t* test). Triangle (▽) indicates a statistically significant difference compared to other immunized groups (p<0.05, Student *t* test).

We also evaluated the combined use of trimeric rHA proteins coupled with the PELC/CpG adjuvant, using either inactivated H5N1 NIBRG-14 virus, or a recombinant adenovirus encoding the full-length HA gene of KAN-1 (H5N1 clade 1) or Anhui (H5N1 clade 2.3.4). Mice immunized with the inactivated NIBRG-14 virus followed by a booster with a trimeric rHA protein elicited slightly higher total IgG titers compared to mice receiving double-NIBRG-14 virus immunizations (Fig. 6A–B). Priming with rAd-HA (Anhui) followed by a booster with a trimeric rHA protein (KAN-1) resulted in the highest anti-Anhui rHA total IgG titer (Fig. 6B). Compared to mice receiving a double-dose of inactivated NIBRG-14, increases of IgG1 subtypes and (to a lesser degree) IgG2a subtypes were observed in mice receiving an initial immunization of inactivated NIBRG-14, rAd-HA (KAN-1), or rAd-HA (Anhui) followed by a booster with KAN-1 or Anhui trimeric rHA proteins (Fig. 6C–D). These combinations produced more balanced Th1 and Th2 responses compared to inactivated NIBRG-14 virus immunization with a trimeric HA protein booster, or two doses of inactivated NIBRG-14.

**Figure 7:**
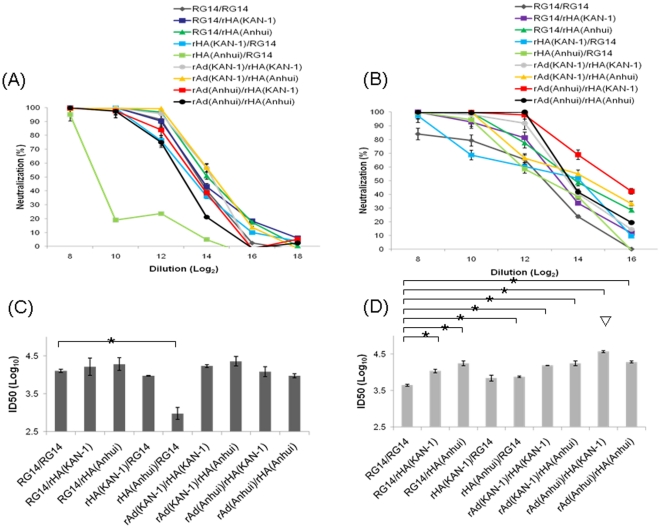
Neutralization against H5 pseudotyped particles in mice receiving combined immunizations. Neutralizing antibody titers in sera were determined using KAN-1 pseudotyped viruses (A, dose-dependent neutralization curves; C, ID50). For neutralization against the KAN-1 strain, mice immunized with NIBRG-14 or rAd (KAN-1) followed by HA (Anhui) had the highest levels of neutralization antibodies; mice immunized with HA (Anhui) followed by NIBRG-14 had the lowest. For neutralization against the Anhui strain (B, dose-dependent neutralization curves; D, ID50), mice immunized with Anhui rAd followed by KAN-1 HA had the highest levels of neutralization antibodies, and mice immunized with two doses of NIBRG-14 had the lowest. Asterisk (*) indicates a statistically significant difference compared to the double-dose of inactivated NIBRG-14 group (p<0.05, Student *t* test). Triangle (▽) indicates a statistically significant difference compared to other immunized groups (p<0.05, Student *t* test).

Neutralization H5pp assays were used to determine neutralizing antibody titers elicited by the use of trimeric rHA proteins in combination with inactivated NIBRG-14 or rAd-HA versus dual-dose NIBRG-14 virus immunizations. According to our results, all of these prime-boost immunization combinations induced dose-independent neutralizing antibody responses against KAN-1 (Fig. 7A) and Anhui (Fig. 7B). The ID-50 values were higher for the inactivated NIBRG-14 virus plus trimeric rHA protein booster than for the two-dose inactivated NIBRG-14 virus immunization against the same or different H5N1 HA clade (Figs. 7C–D). An Anhui rHA protein booster induced more neutralizing antibody titers than a KAN-1 rHA protein booster following inactivated NIBRG-14 virus priming, but the difference was not statistically significant. Compared to two-dose inactivated NIBRG-14 virus immunization, priming with KAN-1 or Anhui rHA protein plus an inactivated NIBRG-14 virus booster resulted in either a slower increase or outright reduction of neutralizing antibody titers against both the homologous (clade 1) and heterologous (clade 2.3.4) strains of H5N1 viruses. Priming with KAN-1 rAd-HA followed by an Anhui rHA protein booster resulted in the highest ID50 values against KAN-1, but the difference was not statistically significant compared to two-dose inactivated NIBRG-14 virus immunization (Fig. 7C). Priming with either rAd-HA (KAN-1 or Anhui) followed by a booster with KAN-1 or Anhui rHA protein elicited stronger neutralizing antibody responses against the H5N1 Anhui clade (2.3.4) than two doses of inactivated NIBRG-14 (Fig. 7D). Priming with Anhui rAd-HA followed by a KAN-1 rHA protein booster resulted in the highest ID50 values against Anhui (Fig. 7D). The use of different HA antigen clades in adenovirus vector-primed and recombinant protein booster immunizations further enhanced neutralizing antibody responses to homologous and heterologous H5N1 virus strains.

## Discussion

Trimeric rHA proteins were prepared using a GCN4-pII leucine zipper sequence, and functionally expressed using a baculovirus-insect cell expression system. Trimeric rHA protein immunogenicity was increased via coupling with a PELC/CpG adjuvant. We found that compared to dual-dose inactivated vaccine immunization, the combined use of trimeric rHA proteins with an inactivated NIBRG-14 vaccine virus or rAd-HA vaccine for prime-boost immunization enhanced antibody responses against homologous and heterologous H5N1 virus strains.

Trimeric HA protein expression of avian and human influenza viruses has been reported for the trimerization sequences of a GCN4-pII leucine zipper [8], [16], [19], [20], [21], [22], [23] and the foldon domain of bacteriophage T4 fibritin [7], [9], [24], [25]. Foldon-derived oligomerization sequences have been described as forming high-molecular-weight oligomers in addition to trimers [7]. Here we used a GCN4-pII leucine zipper sequence that specifically triggers trimerization, rather than GCN4-pIL sequences for dimerization or GCN4-pLI sequences for tetramerization [16], [21]. According to our results, the KAN-1 and Anhui rHA proteins were predominantly trimeric, which is consistent with previous reports on rHA protein expression for the H3N2 [22] and H5N1 viruses [8].

We constructed three truncated forms of trimeric rHA proteins (Tr1, Tr2, Tr3) from the ectodomain HA sequences of two HPAI H5N1 viruses, KAN-1 and Anhui. Our data indicate that (a) Tr1 served as the HA ectodomain (aa 1 to 531); (b) Tr2 (aa 1 to 524) was shorter in length compared to Tr1, with sequences deleted between the transmembrane domain and the bromelain cleavage site; and (c) Tr3 (aa 1 to 517) was the shortest of the trimeric rHA proteins terminated at the bromelain cleavage site. The high-to-low order of HA titers in the three truncated forms was Tr1 > Tr2 > Tr3 (Fig. 2C–D). According to an earlier study, the 14 additional amino acids between Tr1 (terminated at the transmembrane domain) and Tr3 (terminated at the bromelain cleavage site) do not affect neutralizing antibody response in mice; however, Tr3 immunogenicity is greatly reduced by NA co-expression [7]. In contrast, the co-administration of rHA and recombinant NA proteins from the 2009 pandemic H1N1 virus resulted in enhanced HA-specific immune response in ferrets [21].

Monomeric HA proteins are not highly immunogenic in humans [26], [27]. Enhanced rHA protein immunogenicity can be achieved by coupling with adjuvants such as Stimune [8], Titermax [15], Freund [11], Polygen [11], BAY98-7089 [11], and Ribi [7]. For the present study we investigated the adjuvant formulations Alum, CpG, Alum/CpG, PELC, and PELC/CpG to determine their respective effects on trimeric rHA protein immunogenicity. Our results indicate that (a) PECL, an adjuvant recently developed for inactivated NIBRG-14 [17], was more potent than Alum; and (b) CpG resulted in the highest levels of neutralizing antibodies in mice via trimeric rHA immunization. We used 15 µg of trimeric KAN-1 or Anhui rHA protein for mouse immunizations, resulting in the elicitation of anti-H5N1 neutralizing antibody response with a log ID50 value of 2.5 (KAN-1) or 4.2 (Anhui) (Fig. 5C–D). Similar results have been reported for 20 µg of trimeric rHA protein (human cell expression) coupled with Ribi [7] or Alum [28] adjuvant for two-dose mouse immunizations. The corresponding log ID50 values in these studies were 3.3 (Ribi) and 2.1 (Alum). However, a more recent report indicates that only 2 µg of trimeric rHA (expressed in insect cells) coupled with Stimune (a water-in-oil adjuvant) in dual-dose immunizations was sufficient for inducing anti-H5N1 protection in mice [8].

Prime-boost immunization with inactivated A/Vietnam/1203/2004 (clade 1) followed by inactivated A/Indonesia/05/2005 (clade 2.1) has been reported as inducing a potent neutralizing antibody response against heterologous H5N1 virus clades [29], [30]. We therefore examined the combined use of trimeric rHA proteins with inactivated NIBRG-14 vaccine virus as part of a prime-boost immunization strategy to enhance antibody responses against both homologous and heterologous H5N1 virus clades. According to our findings, significantly higher IgG1 titers (as opposed to IgG2a titers) were elicited, suggesting that enhanced Th2 responses were triggered by the inactivated virus-prime and trimeric rHA protein-booster regimen. Following priming with inactivated NIBRG-14, a trimeric Anhui rHA protein (clade 2.3.4) booster resulted in higher neutralizing antibody titers compared to a KAN-1 rHA protein (clade 1) booster, but did not always result in increased IgG titers. Neutralizing antibody responses to homologous and heterologous H5N1 virus clades increased to a greater extent when Anhui trimeric rHA was used as a booster instead of KAN-1 trimeric rHA or inactivated NIBRG-14. The fact that the Anhui strain is more immunogenic than that of the KAN-1 strain was also demonstrated in immunizations using trimeric rHA proteins with any of the investigated adjuvant formulations. The HA protein sequences between KAN-1 and Anhui are highly conserved, with 94.4% amino acid identity or 96.6% amino acid similarity. The region near the 130 loop has been demonstrated as a Ca2 antigenic site in H1HA [31], [32], and several monoclonal antibodies have been isolated to confirm Ca2 antigenicity in H5HA [33], [34]. There are two positive-charge residues (R^139^ and K^140^) near the 130 loop of the receptor-binding domain in KAN-1 HA, but not in Anhui HA; these residues may explain the antigenic differences between the two.

We also found that the combination of trimeric rHA protein and recombinant adenovirus vector as a prime-boost strategy elicited more potent neutralizing antibody responses against homologous and heterologous H5N1 virus strains. Specifically, we investigated KAN-1 rAd-HA or Anhui rAd-HA vaccination followed by a booster of either KAN-1 or Anhui trimeric rHA protein. The sequential HA antigen clade regimen of an Anhui rAd-HA prime and trimeric KAN-1 rHA booster resulted in increased antibody responses in terms of total IgG and neutralizing antibody titers. This result is consistent with other findings that sequential immunization with different H3HA is capable of eliciting significant amounts of broadly neutralizing H3N2 antibodies [35]. However, our results from prime-boost immunizations using different clades of HA antigens to elicit higher neutralizing antibody responses do not agree with previously reported results for the original antigenic sin response of influenza viruses [36]. Further effort is required to investigate the mechanisms through which sequential immunization with different HA antigens can enhance neutralizing antibody response. The combined use of trimeric rHA in prime-boost vaccine regimens may represent an alternative strategy for recombinant H5N1 vaccine development.

## Materials and Methods

### Recombinant H5HA protein construction and purification

Soluble HA proteins were constructed using the HA cDNA sequences of H5N1 A/Thailand/1(KAN-1)/2004 and A/Anhui/1/2005. The A/Thailand/1(KAN-1)/2004 gene was kindly provided by Prasert Auewarakul of Siriraj Hospital at Mahidol University, Thailand. The A/Anhui/1/2005 gene was purchased from Mr. Gene; synthesized sequences were based on the NCBI GenBank accession number DQ371928. The multibasic protease cleavage site between HA1 and HA2, PQRERRRKKRG, was mutated to PQRETRG to retain the uncleaved protein. To obtain trimeric HA proteins, we fused the HA C-terminal sequence with the trimeric GCN4 sequence in front of a thrombin cleavage site, ending with a His-tag to facilitate purification. For large-scale production, Sf9 cells (Invitrogen) were grown in 600 ml SF900-II serum-free medium (Invitrogen) at a cell density of 2×10^6^ cells/ml, then infected with a specific recombinant baculovirus at 3 MOI. Infected cells and culture supernatants were collected 48 h post-infection. Trimeric HA protein expression was determined with SDS-polyacrylamide gels and Western blots using anti-H5HA antibodies (Abcam). Trimeric HA purification was performed with nickel-chelated affinity chromatography (Pierce Protein).

### Cross-linking procedures

Ethylene glycol bis (50 mM) (Sigma) was dissolved in dimethylsulfoxide and diluted into protein solutions at concentrations of 0.25, 1, 2, 5 and 10 mM. Samples were held for 30 min on ice; reactions were stopped by the addition of 50 mM glycine prior to incubation at 37°C for 30 min. Cross-linked samples were analyzed by SDS-PAGE.

### Hemagglutination assays

Purified recombinant HA (100 µg/ml) was serially diluted 2-fold in V-bottom 96-well plates. Equal volumes of 0.05% turkey red blood cells (approximately 4×10^7^ cells/50 µl) (Animal Technology Institute, Taiwan) were added to each well. Plates were covered and held at room temperature for 45 min. HA titers were determined by the reciprocal of the last dilution containing agglutinated turkey red blood cells.

### Inactivated virus and recombinant adenovirus preparations

pENTR (Invitrogen) was used as a transfer vector to create adenoviral vectors containing HA from the H5N1 influenza virus strains A/Thailand/1(KAN-1)/2004 and A/Anhui/1/2005. A full-length coding region of the HA gene was inserted into the transfer vector, followed by positive selection with kanamycin+ LB plate. LR Clonase Enzyme mix (Invitrogen) was used for site-directed recombination between the pENTR vector containing the HA gene and the rAd vector pAd/CMV/V5-DEST (Invitrogen). Following Pac I digestion, the rAd vector was transfected into HEK293A cells (Invitrogen); P1 recombinant adenovirus encoding HA was produced 7∼10 days post-transfection, and then further amplified to produce a P2 virus; virus titer was determined by plaque assay.

H5N1-inactivated NIBRG-14 vaccine, derived from avian influenza virus A/Vietnam/1194/2004 [17], was kindly provided by the Vaccine Research and Development Center (VRDC) of the Taiwan National Health Research Institutes (NHRI). The virus was propagated at the Center in serum-free media (Cesco, Taiwan) using MDCK cells (BCRC, FIRDI, Taiwan) grown in roller bottles. The virus was inactivated by 0.1% formalin at 37°C for 24 hr. HA content of the formalin-inactivated virus was determined by single-radial diffusion assays, using standard antigens and antiserum from NIBSC.

### Adjuvant preparation

We used the CpG ODN sequence 5′-ATC GAC TCT CGA GCG TTC TC-3′ with all phosphorothioate backbones (kindly provided by Ken Ishii of Osaka University). Aluminum phosphate (Alum) (also from the VRDC of the Taiwan NHRI) was given 300 µg doses in acidic media (pH = 6). As previously described, PELC is a squalene W/O/W nanoemulsion adjuvant [17]. Briefly, 120 mg of PEG-*b*-PLACL, 0.8 mL of aqueous solution, and 1.1 mL of oily solution consisting of squalene (Sigma-Aldrich) and Span®85 (Sigma-Aldrich) were emulsified at 6,000 rpm for 5 min and stored at 4°C before use.

### Mouse immunization

Female BALB/c mice (6∼8 weeks old) were immunized with 15 µg purified recombinant HA protein or inactivated NIBRG-14 virus (HA content 0.5 µg) diluted in 50 µl phosphate-buffered saline (PBS, pH 7.4), and mixed with 50 µl of the adjuvant being tested: 10 µg CpG ODN; 300 µg alum with 10 µg CpG; 10% PELC; or 10% PELC with 10 µg CpG. Immunizations were given by intramuscular injection at the beginning of week 0 and at the end of week 3. Blood was collected and serum isolated 14 days following the second immunization. All experiments were conducted in accordance with the guidelines of the Laboratory Animal Center of National Tsing Hua University (NTHU). Animal use protocols were reviewed and approved by the NTHU Institutional Animal Care and Use Committee (approval no. 09733).

### ELISA and anti-HA antibody isotyping

Briefly, 96-well plates were coated with 2 µg/ml of purified trimeric HA protein. After three washes with PBST, samples were blocked with 1% BSA in PBS for 30 minutes at room temperature, followed by three additional washes. The addition of 2-fold serial dilutions of mouse sera was followed by incubation for 1 hr at room temperature, followed by another three washes. Anti-HA IgG antibodies were detected by incubation with peroxidase-conjugated goat anti-mouse IgG antibodies (Abcam) for 1 hr at room temperature. After three washes, TMB substrate was added to develop color, and 2N H_2_SO_4_ was added to stop reactions. Plates were read at 450 nm absorbance; end-point titer was determined as the reciprocal of the final dilution giving an optical of four-fold absorbance of negative control. Anti-HA antibody subclasses were determined by ELISA using anti-IgG1 and anti-IgG2a antibodies.

### H5 pseudotyped particle (H5pp) neutralization assays

Influenza lentiviral pseudotyped viruses were generated as described previously [37]. Briefly, HEK293T cells (BCRC, FIRDI, Taiwan) were co-transfected with pNL Luc E^−^ R^−^ and pcDNA3.1(+) expressing HA from A/Thailand/1(KAN-1)/2004 and A/Anhui/1/2005 strains. *Vibrio cholerae* neuraminidase (6.2 mU/ml; Sigma) was added 24 hr post-transfection to release particles from cells. Culture supernatants were collected and concentrated 48 hr post-transfection. H5pp titer was determined by p24 ELISA (Clontech). Neutralizing antibodies were quantified as reduced luciferase expression level following H5pp transduction in MDCK cells. MDCK cells (4,000 cells/well) were seeded in 96-well plates in 100 µl DMEM. The following day, H5pp (10 ng p24) was incubated with a four-fold serial dilution of anti-sera (starting dilution 1∶64) for 1 hr at 37°C in 60 µl DMEM. Next, 100 µl of fresh medium was added, and 140 µl of the subsequent mixture was transferred to the cells. Fresh medium was added again after 24 hr; luciferase assays were performed 48 hr later via the direct addition of neolite Luciferase substrate (PerkinElmer). Neutralization titers and corresponding standard deviations were determined from neutralization curves using the ID50 program developed by John Spouge of the National Center for Biotechnology Information, National Library of Medicine, US National Institutes of Health.

### Statistical analysis

All results were analyzed using Student's *t* tests, with a *P* value of <0.05 indicating statistical significance. Asterisk (*) and triangle (▽) in the figures indicate a statistically significant difference. All the experiments were performed at least twice with similar results.
